# Transforming growth factor-β1 treatment of oral cancer induces epithelial-mesenchymal transition and promotes bone invasion via enhanced activity of osteoclasts

**DOI:** 10.1007/s10585-013-9570-0

**Published:** 2013-02-02

**Authors:** Jingjing Quan, Moustafa Elhousiny, Newell W. Johnson, Jin Gao

**Affiliations:** 1 Schools of Dentistry, and Medical Science, Griffith Health Institute, Griffith University, Parklands Drive, Southport, Gold Coast, QLD 4222 Australia; 2 School of Medicine and Dentistry, James Cook University, PO Box 6811, Cairns, QLD 4870 Australia; 3 Griffith Health Institute: Population and Social Health Research Group; Molecular Basis of Disease Research Group, Gold Coast Campus, Griffith University, Gold Coast, QLD 4222 Australia; 4 Present Address: Guanghua School and Hospital of Stomatology, Sun Yat-sen University, Guangzhou, People’s Republic of China

**Keywords:** Bone invasion, Osteoclast, Oral squamous cell carcinoma, Transforming growth factor-β1, Epithelial-mesenchymal transition

## Abstract

This study investigates relationships between EMT and bone invasion by OSCC. Three OSCC cell lines, SCC25, HN5, and Tca8113 were artificially induced to display EMT by adding 5 ng/mL of TGF-β1 to culture media for 1–3 days. Cell morphology and phenotypic changes was examined by immunocytochemical staining of CK and VIM. EMT markers, cell-invasion factors, and osteoclast-related molecules were analysed at mRNA, gelatine and protein levels by real-time PCR, gelatine zymography and Western blotting respectively. Mature osteoclasts differentiated from Raw264.7 cells were treated by conditioned medium (CM) of OSCC cells with/without TGF-β1. Immunohistochemistry was performed to validate proteins of CK, VIM, E-cad and Snail1 in OSCC tissue samples with bone invasion. Results showed minimal staining of VIM was found in SCC25 and HN5, while Tca8113 cells stained strongly. EMT markers Twist1 and N-cad were up-regulated; Snail1 and E-cad down-regulated in all cells. Of factors associated with invasion, MMP-2 was unchanged and MMP-9 increased in SCC25 and Tca8113, while MMP-2 was increased and MMP-9 unchanged in HN5. For osteoclast-related molecules, both MT1-MMP and RANKL were up-regulated, while OPG was down-regulated in all cells. CM of OSCC cells pre-treated with TGF-β1 showed to prolong survival of osteoclasts up to 4 days. All target molecules were validated in OSCC samples of bone invasion. These findings suggest that TGF-β1 not only induces EMT to increase the capacity of OSCC for invasion, but also promotes factors which prolong osteoclast survival. TGF-β1 may enhance the ability of MMP2/9 in resorbing bone and favouring invasion of cancer cells.

## Introduction

Bone invasion is one of the most frequent complications of OSCC, especially those arising in the retromolar trigone, buccal sulci, gingiva, floor of mouth and hard palate. The incidence is as high as 56 % of investigated patients [1, 2]. Bone involvement contributes to increased morbidity, higher recurrence and mortality rates [3, 4]. Despite improvements in current treatment modalities—surgery, radiotherapy and adjunctive chemotherapy, cure rates for these patients remain low. Fortunately, there have been considerable advances in understanding the molecular mechanisms of the process of neoplasia, so that the possibility of individualised biotherapies is increasingly recognised. Understanding and interfering with molecules involved in bone invasion may enhance such therapeutic options [5].

When epithelial neoplasms metastasise to bone, a vicious circle is established between the malignant cells themselves, and bone tissue. This also occurs when carcinoma invades bone directly, such as with oral cancer [6]. On the one hand, tumour cells secrete multiple factors to alter the bone environment and induce the formation of osteoclasts; while on the other hand, osteoclasts release growth factors from the bone matrix, which then stimulate tumour growth and further accelerate bone destruction [7, 8]. Of these, TGF-β1, which has been widely studied, is a major bone-derived factor responsible for driving this vicious circle. TGF-β1, along with activins, inhibins, and bone morphogenetic proteins (BMP), are members of the TGF-β super family [9, 10]. For cancer cells, TGF-β1 can become an oncogenic factor to induce proliferation, invasion and immunosuppression during tumour progression [11, 12] and is implicated in bone metastasis from several solid tumours. Mohammad et al. showed that the inhibitor of TGF-β receptor I (TβRI) kinase, effectively reduced osteolytic lesions and tumour burden in mice with malignant melanoma [13]. Ganapathy et al. demonstrated that two types of TGF-β pathway antagonists (1D11 and LY2109761) significantly decreased metastasis to lung and bone of nude mice, injected with cells of human basal-like breast cancer [14]. As for bone invasion by OSCC, a recent study found that TβRI was expressed by 18 of 21 patients with gingival SC, and the inhibitor of TβRI greatly reduced the bone destruction caused by OSCC cells in vitro [15]. Additionally, Prime et al. [16] observed that the OSCC cell lines, which were resistant to inhibitory effects of TGF-β1, formed significantly more primary tumours, with high incidence of mandibular invasion, when injected through the floor of the mouth of athymic mice.

In the present study, we found that conditioned medium (CM) of osteoblasts induced gene expression level changes of OSCC cells [17], for example, Twist1, was up-regulated in these cells after such treatment. The expression of MMP-2 protein level was increased, while MMP-9 was decreased. Furthermore, immunohistochemical staining of Twist1, MMP-2 and MMP-9 was observed in clinical samples of OSCC patients with bone invasion. We speculate that these changes in gene expression may be caused by growth factors in the CM. Since TGF-β1 was present in significant amounts in the bone microenvironment, and proven to induce EMT in various epithelial cells in vitro [12], we attempted to determine the effects of TGF-β1 on OSCC cells and the way they interact with osteoclasts.

## Materials and methods

### Reagents and antibodies

Recombinant TGF-β1 was purchased from R&D Systems (Minneapolis, USA). DMEM, foetal bovine serum (FBS), trypsin–EDTA, anti-CK (anti-pan cytokeratin), anti-VIM and anti-E-cad were purchased from Invitrogen. Anti-Twist1, anti-Snail1, anti-MT1-MMP, anti-tissue inhibitors of MMP (TIMP1), anti-TIMP2 and anti-OPG were bought from Santa Cruz Biotechnology; while anti-RANKL and anti-α-Tubulin were from Abcam. Anti-N-cad was purchased from Cell Signalling. Primary antibodies were either mouse or rabbit against human. Secondary antibodies, goat anti-mouse IgG and goat anti-rabbit IgG, were from Bio-Rad Laboratories.

### Indirect co-cultures

OSCC cell lines of SCC25, HN5 and Tca8113 were kindly supplied by Associate Professor Nick Saunders (The University of Queensland, Australia), Professor Ming Wei (Griffith University, Australia) and Professor Qian Tao (Sun Yat-sen University, China) respectively. These cells were routinely maintained in DMEM containing 10 % FBS. Human foetal osteoblasts (hFOB) were obtained from American Type Tissue Collection (ATCC, Rockville, USA), and grown in DMEM/F12 with 10 % FBS plus 300 μg/mL geneticin (G418) at 34 °C in an incubator. CM [17] was collected from OSCC cell lines SCC15 and SCC25, and from hFOB, and used for indirect co-culture: OSCC cells were treated with CM of hFOB cells for 48hs. To block the endogenous TGF-β1 secreted by hFOB cells, TGF-β inhibitor (SB431542, 188 nM, Sigma) was used in CM of hFOB to treat OSCC cells for 48 h. To examine the effects of TGF-β1 on OSCC cells, equal numbers of SCC25, HN5 and Tca8113 cells were plated in serum-free medium, starved for 12 h, and then treated with TGF-β1 (5 ng/mL) for 0, 1–3 days. Culture medium was changed daily.

### Immunocytochemistry

After the treatment with TGF-β1 for 0–3 days, OSCC cells were fixed with 70 % ethanol for 10 min and permeabilized by 0.1 % Triton X-100 for 5 min. Non-specific binding of the antibodies was avoided by blocking with 5 % BSA in PBS for 30 min, followed by incubation with primary antibodies of CK (1:50) and VIM (1:50) overnight at 4 °C, and then with secondary antibodies for 1 h at 37 °C. Non-immune serum instead of the primary antibody was used as negative controls. Three washes with PBS were applied between each step of antibody incubation. Sites of binding were visualized using liquid diaminobenzidine (DAB) + substrate + chromogen system (DAKO), counterstained with Mayer’s haematoxylin, and photographed by a Nikon OXM1200 digital camera with the Act-1 program. Immunostaining intensity was scored according to percentage of tumour cells with positively-stained: designated as + where <20 % of cells were stained; ++ where <40 % were stained; +++ where >60 % were stained.

### Real-time PCR

Total RNA was isolated from OSCC cells before and after TGF-β1 treatment using the PureLink RNA Mini Kit (Invitrogen, USA), and reverse transcribed to cDNA using the iScript cDNA Synthesis Kit (Bio-Rad) based on the manufacturer’s instructions. Quantitative gene analysis was performed for Twist1, Snail1, E-cad, N-cad, MMP-2, MMP-9, TIMP-1, TIMP-2, MT1-MMP, RANKL and OPG by using EXPRESS SYBR GreenER qPCR Supermix Universal Kit (Invitrogen, USA) and the icycler iQ5 Real-time PCR system (Bio-Rad, USA). The data were normalized to the internal control, GAPDH to obtain ΔCt. Finally fold-change of genes of interest relative to untreated samples was reported by 2^−ΔΔCt^ method. Primers used in this study are listed in Table 1.

**Table 1:** Primer Sequences Used in Real-time PCR

Genes	Primers	Length (bp)
Twist1	Forward: 5′-TGTCCGCGTCCCACTAGC-3′ Reverse: 5′-TGTCCATTTTCTCCTTCTCTGGA-3′	63
Snail1	Forward: 5′-TGCAGGACTCTAATCCAAGTTTACC-3′ Reverse: 5′-GTGGGATGGCTGCCAGC-3′	71
E-cad	Forward: 5′- GAACAGCACGTACACAGCCCT-3′ Reverse: 5′-GCAGAAGTGTCCCTGTTCCAG-3′	76
N-cad	Forward: 5′- GACGGTTCGCCATCCAGAC-3′ Reverse: 5′-TCGATTGGTTTGACCACGG-3′	66
MMP-2	Forward: 5′-GACATACATCTTTGCTGGAGAC-3′ Reverse: 5′-TTCAGGTAATAGGCACCCTT-3′	180
MMP-9	Forward: 5′-CTTCACTTTCCTGGGTAAG G-3′ Reverse: 5′-CACTTCTTGTCGCTGTCAAA-3′	105
TIMP-1	Forward: 5′-GGAGAGTGTCTGCGGATACTTC-3′ Reverse: 5′-GCAGGTAGTGATGTGCAAGAGTC-3′	100
TIMP-2	Forward: 5′-ACCCTCTGTGACTTCATCGTGC-3′ Reverse: 5′-GGAGATGTAGCACGGGATCATG-3′	129
MT1-MMP	Forward: 5′-CCTTGGACTGTCAGGAATGAGG-3′ Reverse: 5′-TTCTCCGTGTCCATCCACTGGT-3′	146
RANKL	Forward: 5′-CAGAAGATGGCACTCACTGCA-3′ Reverse: 5′-CACCATCGCTTTCTCTGCTCT-3′	203
OPG	Forward: 5′-GGAACCCCAGAGCGAAATACA-3′ Reverse: 5′-CCTGAAGAATGCCTCCTCACA-3′	225
GAPDH	Forward: 5′-TGCACCACCAACTGCTTAGC-3′ Reverse: 5′-GGCATGGACTGTGGTCATGAG-3′	87

### Gelatine zymography

Equal numbers of OSCC cells of SCC25, HN5, and Tca8113 were planted in 6-well plates with the density of 10^6^ cells per well. CM from OSCC cells before and after TGF-β1 treatment was used for Gelatine Zymography. CM of HT 1,080 cells served as the positive control. 20 μL of each sample was mixed with non-reducing sample buffer (62.5 mM Tris–HCl, pH 6.8; 4 % SDS; 25 % glycerol; 0.01 % Bromophenol Blue) and electrophoresed on 10 % precast denaturing SDS polyacrylamide gels with gelatine (Bio-Rad Lab, CA, USA). Gels were washed in the commercial renature solution (2.5 % Triton X-100) for 40 min at room temperature and incubated in development solution (50 mM Tris; 200 mM NaCl; 5 mM CaCl_2_; 0.02 % Brij-35) at 37 °C for 40 h. Finally, gels were stained with Coomassie Brilliant Blue R-250 (Sigma, USA) for 1 h at room temperature and progressively destained until clear bands appeared against the blue background.

### Western blotting

Total protein was extracted from OSCC cells before and after TGF-β1 treatment using lysis buffer (Thermo scientific, USA). The protein concentration was determined using a BCA Protein Assay Kit (PIERCE, USA). 40 μg of protein was subjected to SDS–PAGE with 10 % polyacrylamide gels. Proteins were transferred to PVDF membranes, and blocked with 5 % non-fat dry milk in Tris-buffered saline (TBS) for 1 h at room temperature. The membranes were then incubated with primary antibodies of Twist1 (1:200), Snail1 (1:200), E-cad (1:200), N-cad (1:100), TIMP-1 and TIMP-2 (1:200), MT1-MMP (1:200), RANKL (1:500), OPG (1:200) and α-Tubulin (1:3,000) overnight at 4 °C, washed twice and incubated with horseradish peroxidase-conjugated (HRP) secondary antibodies for 1 h at room temperature. The protein bands were detected by SuperSignal WestPico Chemiluminescent Substrate (Thermo scientific) and visualised using the VersaDoc-MP Imaging Systems (Bio-Rad).

### Osteoclasts differentiation from RAW264.7 cells

Mature osteoclasts were generated from the murine macrophage cell line of RAW264.7, which was kindly given by Dr Stephen Hamlet (Griffith University). RAW264.7 cells were cultured in DMEM with 10 % FBS at 37 °C in a humidified 5 % CO_2_ atmosphere. To obtain osteoclasts, these cells were seeded in a 96-well plate at a density of 1.25 × 10^4^ cells/well, and supplemented with 50 ng/mL of recombinant mouse RANKL (R&D Syetems, USA) on day 1 and day 3. Mature osteoclasts were observed on day 5. Afterwards, the entire culture medium was changed into CM of OSCC cells pre-treated with or without TGF-β1 (5 ng/mL). After 4 days’ treatment, TRAP staining (STrACP, Nanjing Jiancheng, China) was performed and photographed using a Nikon OXM1200 digital camera with the Act-1 program.

### Validation of EMT related markers in clinical samples

To validate proteins of CK, VIM, E-cad and Snail1, the archival blocks from 12 patients whose OSCC showed invasion of bone were randomly examined. The Human Research Ethical Clearance was granted by both Griffith University and Queensland Health prior to the commencement of the study. Serial tissue sections (5 μm thickness) were dewaxed, rehydrated and treated with 0.3 % hydrogen peroxide in PBS. Antigen retrieval was performed in 0.2 % citrate buffer (pH 6.0) by heating sections in a microwave oven (2 × 4 min). After non-specific binding was blocked with 5 % BSA in PBS for 30 min, sections were incubated with primary antibodies of CK (1:50), VIM (1:50), E-cad (1:80) and Snail1 (1:100) overnight at 4 °C. Sections were then treated with the anti-mouse/rabbit secondary antibodies (Envision + Systems) for 30 min, followed by DAB detection solution (Dako, Botany, Australia) for a few minutes at room temperature. Primary antibodies were replaced by non-immune serum as negative controls. Sections were counterstained with Mayer’s haematoxylin, dehydrated, and mounted with DPX (BDH Laboratory, Poole, England). The final results were visualized by a light microscopy and photographed using an Olympus Bx60 digital camera with the CellSens software.

### Statistical analysis

Data analysis was performed using SAS program (SAS version 8.1, USA). A paired Student t test was used to compare two means. A *p* value of less than 0.05 was regarded as significant.

## Results

### Indirect co-cultures between osteoblasts and OSCC cells

**Fig. 1:**
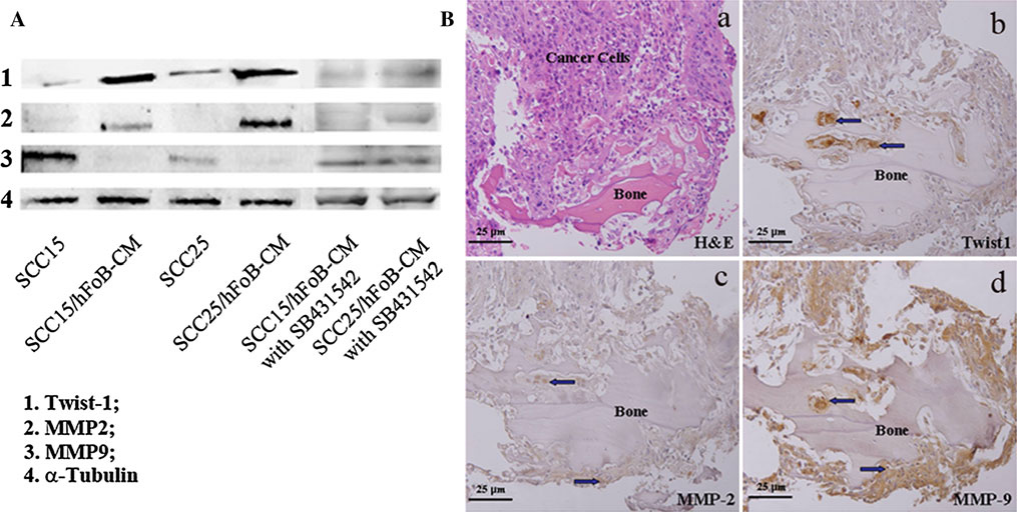
Results of the indirect co-culture between hFOB and OSCC cells. **a** Western blotting shows that Twist1 expression is up-regulated after the treatment with CM from hFOB cells. The expression of MMP-2 is increased, while MMP-9 decreases in all OSCC cells. Expression of Twist-1, MMP-2 by OSCC cells was reduced, while MMP-9 was increased after the blockage of TGF-β1 by using SB431542. **b** H&E staining shows an infiltrative pattern of bone invasion with osteoclasts accumulating in resorption lacunae (**a**). Weak immunohistochemical staining of Twist1 shows in the cytoplasm of OSCC cells, but strongly stained in osteoclasts (**b**). MMP-2 is weakly expressed in OSCC cells and osteoclasts (**c**), while MMP-9 is clearly localized within the cytoplasm of OSCC cells, especially in the nuclei of osteoclasts (**d**)

Results showed that Twist1 expression was up-regulated in OSCC cells after the treatment with CM from cells of hFOB. MMP-2 was increased while MMP-9 was decreased in all OSCC cells. To verify the effect of TGF-b secreted by hFOB in the co-cultures, hFOB was pretreated with the inhibitor of TGF-b (SB431542) followed by the co-culture. It was found the reduction of expressions of Twist-1 and MMP-2, but slightly increased MMP-9, which suggested bioefficiency of the inhibitor (Fig. 1a). Immunochemical staining of these molecules was observed in 12 clinical samples of OSCCC patients with bone invasion (Fig. 1b): H&E staining showed an infiltrative pattern of bone invasion with tumour cells invading into the bone, and osteoclasts accumulated in resorption lacunae. Faint staining of Twist1 was noted in the cytoplasm of OSCC cells, but strongly in osteoclasts. MMP-2 was weakly expressed in OSCC cells and osteoclasts, while MMP-9 was clearly localized within the cytoplasm of OSCC cells and especially in the nuclei of osteoclasts.

**Fig. 2:**
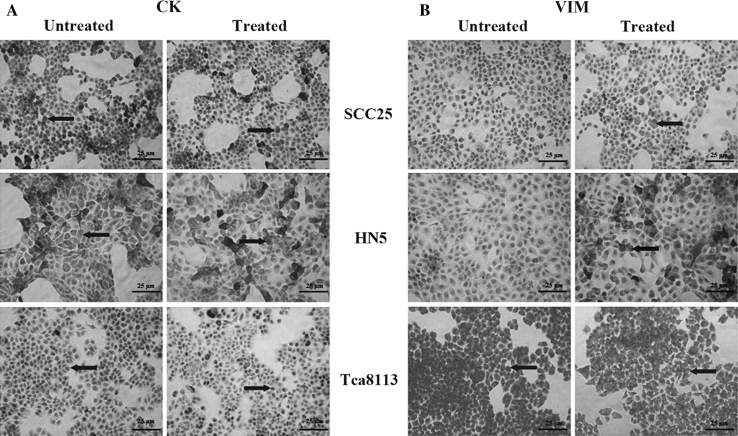
Immunohistochemical staining of CK and VIM in OSCC cells. **a** Similar staining patterns are visualised at each time point: CK staining has no change in the epithelial OSCC cells (*arrows*, *bar* = 25 μm); weak staining of VIM is shown in SCC25 and HN5, while VIM is strongly stained in Tca8113 cells before and after the treatment (*arrows*, *bar* = 25 μm). Results represent three independent experiments. **b** Summary of the immunohistochemical staining results

The cell morphology of OSCC remained no change, neither was in the staining intensity of cytokeratin changed (Fig. 2a). A summary of the staining results is shown in Fig. 2b. However, weak staining of VIM was found in SCC25 and HN5 following the treatments with TGF-β1. Same staining of VIM was also found in Tca8113 cells before and after the treatments (Fig. 2b).

### mRNA level changes of selected genes after treatment with TGF-β1

**Fig. 3:**
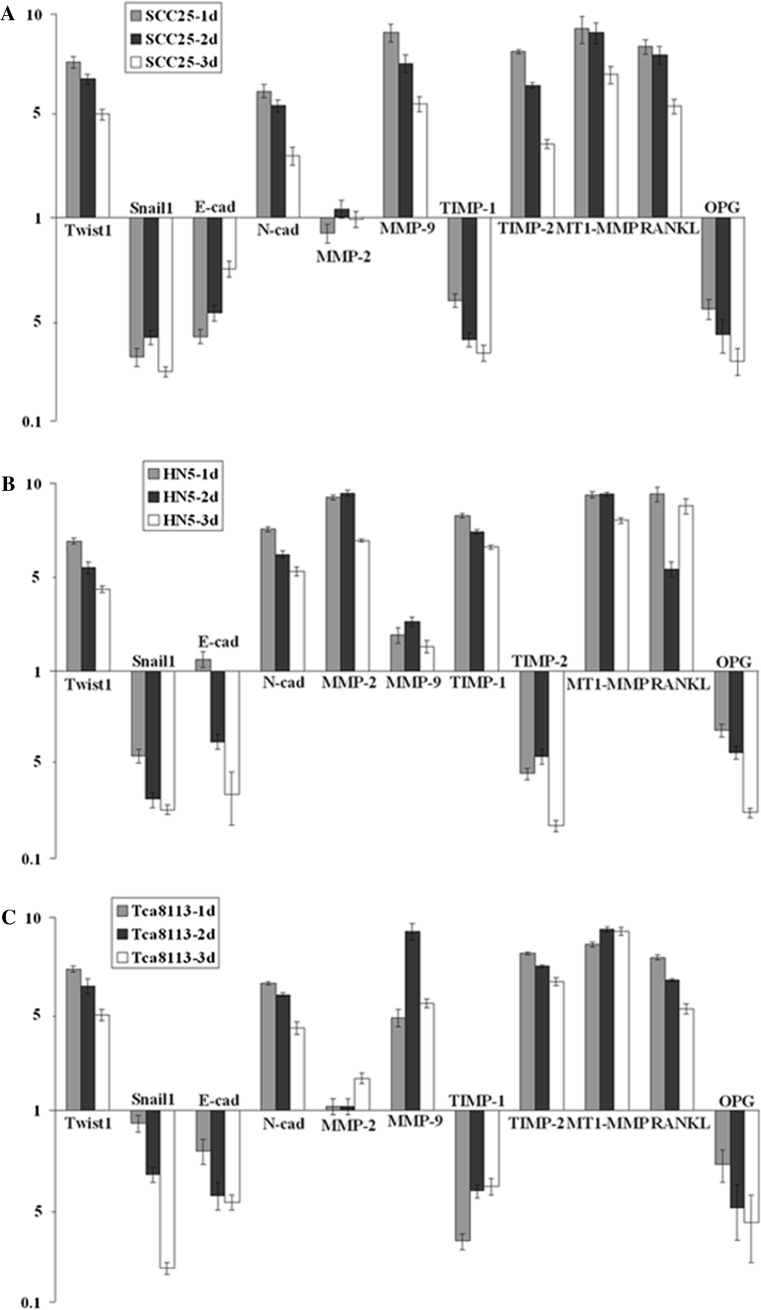
Real-time PCR in three OSCC cell lines (**a** SCC25, **b** HN5, **c** Tca8113) before and after treatments with TGF-β1. The expression of EMT markers, Twist1 and N-cad, are shown to have increased expression, while Snail1 and E-cad are down-regulated in all types of cells following the treatments with TGF-β1. The cell invasion factor, MMP-2, is not affected in SCC25 and Tca8113 cells, but the expression of MMP-2 is induced in HN5 cells. Conversely, MMP-9 is not affected in HN5 cells, while it is up-regulated in SCC25 and Tca8113 cells. Moreover, TIMP-1 is suppressed in SCC25 and Tca8113 cells, while the expression of HN5 cells is increased. On the other hand, TIMP-2 is induced in SCC25 and Tca8113 cells, but is suppressed in HN5 cells. For osteoclast-related molecules, both MT1-MMP and RANKL are induced while OPG is suppressed in all cells tested. Data are shown as mean ± SD of three independent experiments

Real-time PCR was utilized to examine mRNA levels of selected genes. The EMT markers, Twist1 and N-cad, were shown to have increased expression, while Snail1 and E-cad were down-regulated in all cells after treatment with TGF-β1 (Fig. 3a–c). The cell invasion factor MMP-2 was not affected in SCC25 and Tca8113 cells, while it was induced in HN5 cells. Conversely, MMP-9 was un-affected in HN5, but was up-regulated in SCC25 and Tca8113 cells. Furthermore, TIMP-1 was decreased in SCC25 and Tca8113 cells, while the expression of HN5 was increased. TIMP-2 was induced in SCC25 and Tca8113, but suppressed in HN5 cells. For osteoclast-related molecules, the expression of both MT1-MMP and RANKL was increased, but OPG was suppressed in all cells.

### Changes of zymogenic activity of MMP-2 and MMP-9 after treatments with TGF-β1

**Fig. 4:**
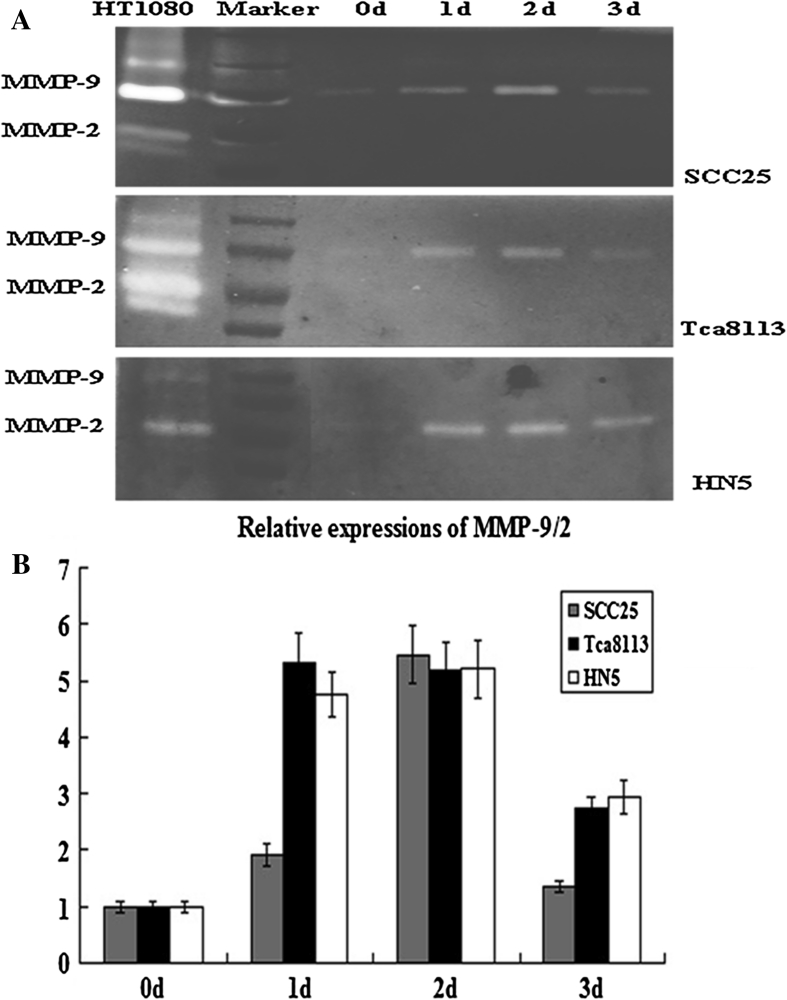
Gelatin Zymography in OSCC cells before and after treatment with TGF-β1. **a** TGF-β1 mediates the activities of MMP-9 in both SCC25 and Tca8113 cells, while the activity of MMP-2 is also increased in HN5 cells. **b** The intensity of bands shown in the zymograph is converted to the figure using semi-quantitative histodensitometry analysis. The results are averaged in three independent experiments

Zymography was used to detect the zymogenic activities of MMP-2/-9 (Fig. 4a). Results showed that TGF-β1 increased the activities of MMP-9 in SCC25 and Tca8113 cells, and the activities of MMP-2 in HN5 cells (Fig. 4b).

### Expression of protein changes of targeted genes after treatment with TGF-β1

**Fig. 5:**
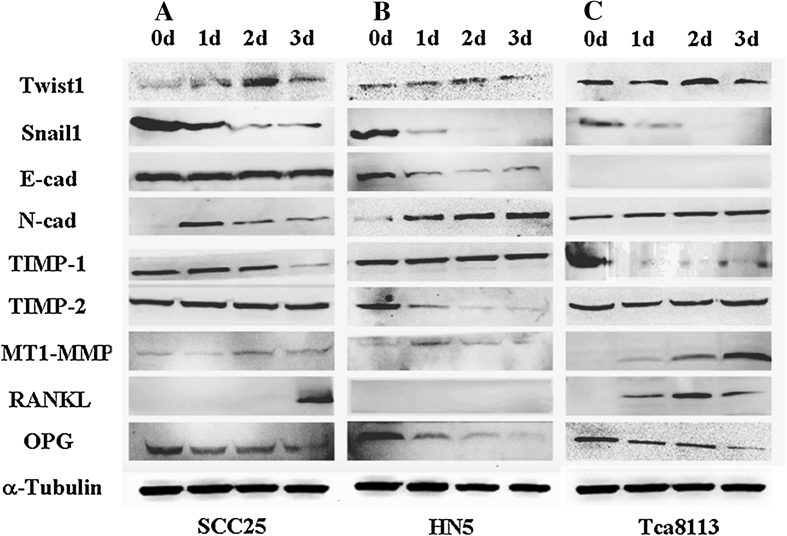
Western blotting analysis of three OSCC cell lines (**a** SCC25, **b** HN5, **c** Tca8113) before and after treatment with TGF-β1. For EMT markers, Twist1 expression is increased while Snail1 is decreased in all these cells. E-cad is slightly suppressed in SCC25 while it is dramatically suppressed in HN5, and not detected in Tca8113 cells. However, expression of N-Cad demonstrates to be switched on in all types of OSCC cells. TIMP-1 is *down-regulated*, but TIMP-2 has no change in both SCC25 and Tca8113 cells. While TIMP-1 is not affected, TIMP-2 is suppressed in HN5 cells. For osteoclast-related molecules, MT1-MMP is *up-regulated* in all OSCC cells. RANKL is induced in SCC25 and Tca8113 cells, while it is not detected in HN5 cells. Expressions of OPG are decreased in all OSCC cells. These results are representative of three independent experiments

Western blotting was performed to validate expressions of TGF-β1 targeted genes at the protein level. Comparing with results of real-time PCR, the expression pattern was almost the same with a few differences (Fig. 5a–c). For EMT markers, Twist1 was increased while Snail1 was decreased in all OSCC cells. E-cad was slightly suppressed in SCC25 cells while it was dramatically suppressed in HN5 cells, but not detected in Tca8113 cells. N-cad was found to be induced in all cells tested. TIMP-1 was down-regulated and TIMP-2 was un-changed in both SCC25 and Tca8113 cells; while TIMP-1 was un-affected and TIMP-2 was suppressed in HN5 cells. For osteoclast-related molecules, MT1-MMP was up-regulated in all cells. Furthermore, RANKL was induced in SCC25 and Tca8113 cells, while it was not detected in HN5 cells. Expressions of OPG were decreased in all OSCC cells.

### CM of OSCC cells treated with TGF-β1 prolonged the survival of mature osteoclasts

**Fig. 6:**
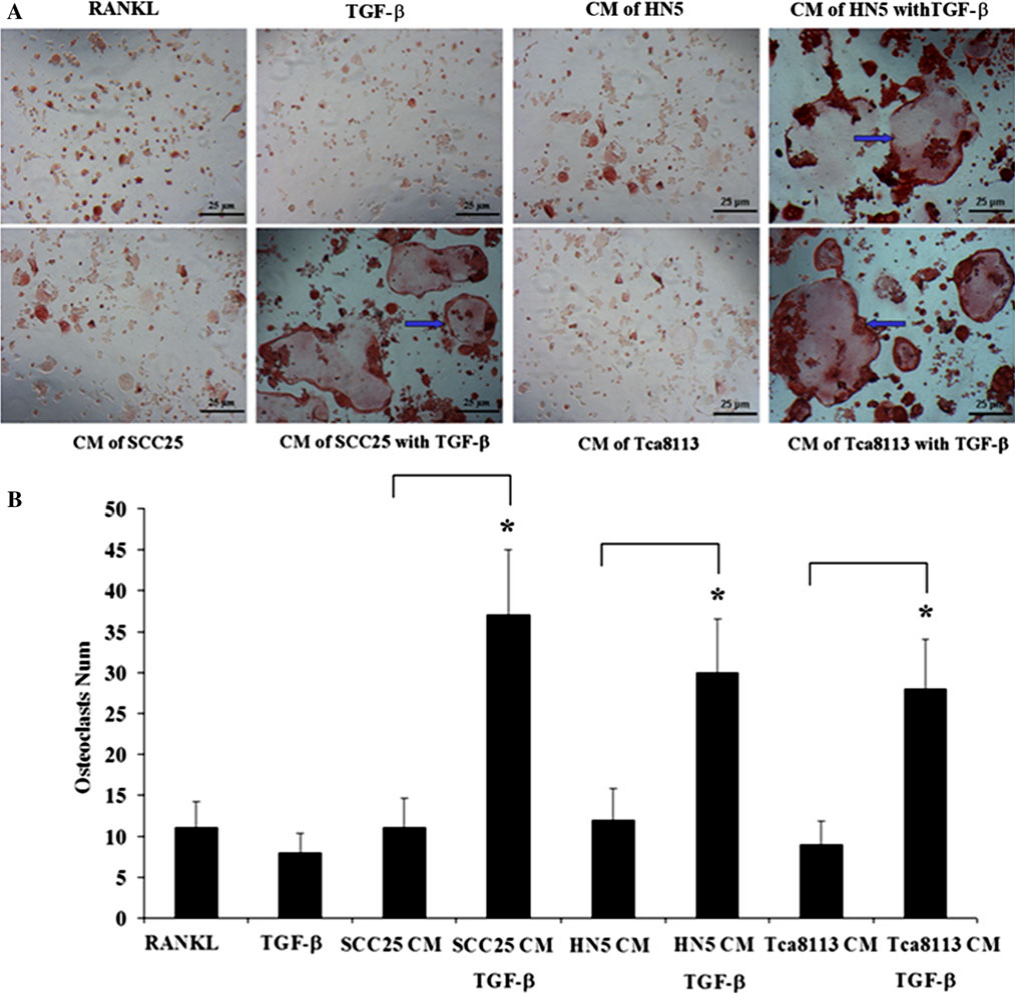
TRAP staining of the mature osteoclasts generated from Raw264.7 cells. **a** The *positive control* group of osteoclasts (with continual RANKL treatment) become apoptotic on day 4 (TRAP, *bar* = 25 μm). TGF-β1 (5 ng/mL) also directly induces the apoptosis of mature osteoclasts on day 4 (TRAP, *bar* = 25 μm). Comparing with CM of OSCC cells without TGF-β1, CM of OSCC cells pre-treated with TGF-β1 may prolong the survival of mature osteoclasts up to 4 days (*arrows*, TRAP, *bar* = 25 μm). **b** Numbers of osteoclasts are counted with 4 fields randomly selected. Data are shown as M ± SD of three independent experiments (*, *p* < 0.05)

Mature osteoclasts generated from the murine macrophage cell line, RAW264.7, were treated with CM of OSCC cells with or without TGF-β1. Since our earlier results had shown significant changes of EMT markers on days 1 and 2, CM of OSCC cells were here treated with TGF-β1 for 24 or 48 h. Similar results were observed on each day as follows: comparing with CM from OSCC cells without TGF-β1, CM from OSCC cells pre-treated with TGF-β1 prolonged the survival of osteoclasts up to 4 days (Fig. 6a–b). Osteoclasts with continual RANKL treatment became apoptotic on day 4, TGF-β1 (5 ng/mL) also induced the apoptosis of mature osteoclasts on day 4 (Fig. 6a–b).

### Validation of targeted molecules in human OSCC tissues with bone invasion

**Fig. 7:**
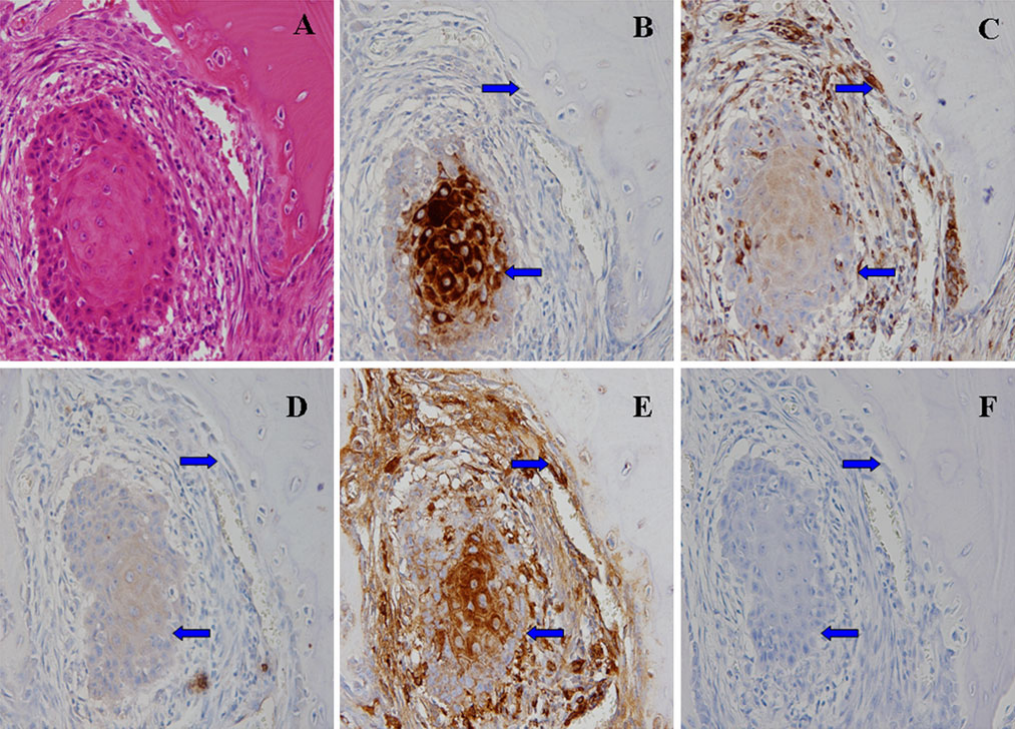
Validation of targeted molecules in OSCC tissue with bone invasion using the immunohistochemical analysis. **a** The H&E staining shows the infiltrative pattern of bone invasion by OSCC into the bone tissue. **b** Immunohistochemistry shows that CK is strongly expressed in the cytoplasm of OSCC cells at the centre of tumour, while *weak staining* is found in cancer cells at the front of bone resorption sites. **c** The VIM is weakly stained within the centre of tumour, but strongly stained in OSCC cells at the front of bone resorption sites. **d** Cytoplasmic expression of E-cad is weakly at the centre of tumour, but no staining in OSCC cells is shown at bone resorption sites. **e** The expression of Snail-1 is strongly present throughout the cytoplasm of all OSCC cells. **f** The *negative control* shows no staining in OSCC cells

The H&E staining on archived OSCC tissue sections obtained from 12 patients with bone invasion showed an infiltrative pattern, and cancer cells invaded into the bone tissue (Fig. 7a). Using immunohistochemistry, it was found that CK was strongly expressed in the cytoplasm of OSCC cells, while VIM was weakly stained within the cytoplasm of OSCC cells (Fig. 7b–c). For E-cad, weak cytoplasmic expression was found in OSCC cells (Fig. 7d). Stronger cytoplasmic expression of Snail1 was observed in cytoplasm of OSCC cells (Fig. 7e). Control sections were negatively stained (Fig. 7f).

## Discussion

TGF-β is well known to be a key initiator of EMT, which can induce artificial EMT of normal epithelial cells as well as of malignant cells [12, 18]. In our present study, we observed that cell morphology in these OSCC cells cultures was not changed, most cells remaining polygonal during 3 days’ treatment with TGF-β1. This is consistent with our earlier studies in which morphological evidence of EMT took several days longer to become manifest [19]. For these EMT markers, typical “cadherin switching”, a term referring to the ability of E-cad expression and activity to switch on the expression of N-cad [20], was shown in all cell lines tested. Twist1 expression was increased while Snail1 was decreased in all cells after being treated with TGF-β1. Both Twist1 and Snail1 are nuclear transcription regulators of EMT which interact with each other, not always in a consistent fashion [21–23]. For example, Twist1 was found to act upstream from Snail and induce EMT-like transformation in a mouse xenograft model with human breast cancer [21]; while a comparison of benign and malignant pheochromocytoma suggested that Snail target on Twist promoting malignant transformation [22]. Whilst the reasons for these different interpretations arising in different experimental situations are currently unknown, the present study, consistent with previous co-cultures, and the increased expression of Twist1 suggesting TGF-β1 be the active factor in CM which initiates EMT.

The gelatinases MMP-2/-9 are associated with EMT to increase tumour invasion and metastasis [24]. We previously reported that expression of MMP-2/-9 in OSCC cells was, in relation to EMT, triggered by TGF-β1 [19]. MMP-2 and MMP-9 are secreted but rapidly become inhibited by the specific endogenous inhibitors of TIMP-1 and TIMP-2 [25]. In the present study, TIMP-1 expression decreased and TIMP-2 increased in both SCC25 and Tca8113 cells. Conversely, MMP-2 expression was up-regulated and MMP-9 was not changed, while TIMP-1 expression was up-regulated and TIMP-2 was down-regulated in HN5 cells. The increased expression of MMP-9 or MMP-2 in these OSCC cells suggested similar responses to TGF-β as shown before [26, 27]. This may establish an autocrine loop: both MMP-2 and MMP-9 can mediate the cleavage of latent TGF-β complexes [28]. Moreover, an autocrine regulation of MMP and TIMP was confirmed that endogenous MMP-9 was inhibited by TIMP-1, and TIMP-2 was the primary inhibitor of MMP-2. It is currently unknown whether this regulation is direct or indirect action of TGF-β. Indeed, many signalling pathways have been involved in the regulation of TGF-β, MMP and TIMP family members, which will be explored in future studies.

Osteoclast-related factors not only include molecules which induce the formation of osteoclasts, but also those proteolytic enzymes which help to degrade the non-mineralized osteoid [29]. The gelatinases MMP-2 and MMP-9 participate in the recruitment of osteoclast precursors and the differentiation of osteoclasts during the development and growth of normal tissues [30]. In addition to the gelatinase activities of MMP-2/-9, MT1-MMP functions as a sheddase, releasing non-ECM substrates such as RANKL [31]. A recent report demonstrated that MT1-MMP derived from prostate cancer cells enhanced their migration through an autocrine pathway of MT1-MMP/RANK/RANKL [32]. The present study has shown that MT1-MMP was increased in OSCC cells after being treated with TGF-β1. To establish whether MT1-MMP has similar autocrine effects in OSCC cells as demonstrated for prostate cancer cells, we analysed the expression of RANKL and OPG and found the expression of RANKL was increased, but OPG was decreased in OSCC cells. Therefore, the suppression of OPG expression tipped the ratio of RANKL to OPG in favour of RANKL, which may lead to enhance osteoclastogenesis resulting in bone resorption [33].

**Fig. 8:**
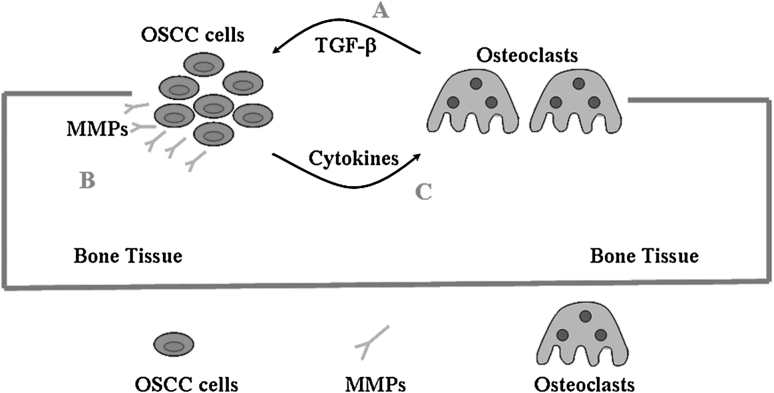
Pathways involve in the progression of bone invasion by OSCC. **a** Osteoclasts play the main role to degrade the bone matrix, growth factors such as TGF-β are released from the bone components. **b** TGF-β may induce EMT of OSCC cells and promote expressions of MMPs, which directly facilitate invasion of cancer cells through stromal tissues or micro-cavities of bone marrow within the bone. **c** TGF-β may also promote cytokines such as osteoclasts-related factors, which activate more osteoclasts and prolong their survival favouring the invasion of cancer cells

We next wished to investigate the effects of molecules released by OSCC cells on osteoclast behaviour (Pathway C, Fig. 8). Unexpectedly, when CM of OSCC cells was added into cultures of Raw264.7 cells, we could not see osteoclast formation (data not shown). Thereafter, we firstly obtained mature osteoclasts from Raw 264.7 cells, and treated them with CM of OSCC cells grown with or without TGF-β1. Our results showed that CM from cells pre-treated with TGF-β1 prolonged osteoclast survival up to 4 days compared with CM without TGF-β1. With continued RANKL treatment, mature osteoclasts became apoptotic on day 4. TGF-β1 (5 ng/mL) also induced apoptosis of these osteoclasts. Thus, our results indicated that factors in OSCC cells treated by TGF-β1 might block its apoptotic effects, and promote survival of osteoclasts. Cytokines induced by TGF-β1 may include pathways of apoptosis, such as caspase or BCL family members, which needs to be further investigated.

The question of whether changes in the fibroblast-shaped neoplastic cells are necessary for these to acquire osteomimetic characteristics remains [34, 35]. Davies et al. [36] found that transfection of TGF-β1 into a rat keratinocyte cell line caused changes in cell morphology from polygonal to spindle: these cells subsequently formed tumours in nude mice, and increased local bone resorption. Takayama et al. [37] reported that TGF-β1 caused EMT of SCCVII cells, and they found EMT-like changes through resected human mandibles with gingival SCC, in which immunohistochemical staining of E-cad was weak in tumour mass-margin comparing with the tumour mass-centre. Although changes of cell morphology (indicative of EMT) was not observed during the 3 day treatments with TGF-β1, OSCC cells coordinated the survival of osteoclasts. Further validation of molecules in OSCC tissue samples found stronger staining of CK and Snail1, while weaker staining of VIM and E-cad, which are consistent with Takayama’s results. Although these findings are indirect observation, which are not substantial, they suggest that partial EMT may exist in the bone invasive progression of OSCC. Whether morphologic and phenotypic changes of malignant keratinocytes are necessary for bone invasion is the focus of future work [38].

## Abbreviation

OSCC: Oral squamous cell carcinoma
EMT: Epithelial-mesenchymal transition
CM: Conditioned medium
TGF-β1: Transforming growth factor-β1
MMP-2: Matrix metalloproteinase-2
MMP-9: Matrix metalloproteinase-9
CK: Cytokeratin
VIM: Vimentin
E-cad: E-cadherin
N-cad: N-cadherin
MT1-MMP: Membrane type 1 matrix metalloproteinase
TIMP-1: Tissue inhibitor of matrix metalloproteinase-1
TIMP-2: Tissue inhibitors of matrix metalloproteinase-2
RANKL: Receptor activator of nuclear factor (NF)-κΒ ligand
OPG: Osteoprotegerin
TRAP: Tartrate-resistant acid phosphatase
